# Neurocognitive function among individuals with problematic social media use

**DOI:** 10.3389/fpsyt.2026.1825888

**Published:** 2026-05-18

**Authors:** Inbar Almog, Yaniv Mama, Aviv M Weinstein

**Affiliations:** Psychology Department, Ariel University, Ariel, Israel

**Keywords:** Compulsivity, delay discounting, excessive social media use, go/no-go, impulsivity, WCST

## Abstract

**Background:**

With the development of technology and the internet, social networks gained momentum quickly and play a central role in daily activities. Despite this, there is a public health concern over excessive or problematic social media use. There is also a debate whether excessive social media use should be considered as a behavioral addiction characterized by impulsivity or an impulse control disorder characterized by compulsivity. The goal of this study is to use neurocognitive tasks to investigate impulsivity and compulsivity among excessive social media users compared with non-excessive users.

**Method:**

The study included 79 participants (age range 18 to 37), divided into two groups: 34 participants who excessively use social media (Mean Age = 23.03, SD = 2.71) and 45 participants who do not excessively use social media (Mean Age = 25.47, SD = 4.3). Participants filled out a demographic questionnaire, questionnaires on social media use, impulsivity, compulsivity, anxiety, and depression. They performed computerized cognitive tasks: GO/NO-GO (with Facebook and traffic sign pictures), Experimental Delay Discounting (EDT), and the Wisconsin Card Sorting Test (WCST).

**Results:**

Excessive users of social media exhibited a lower ability to delay gratification on the EDT, indicating impulsivity. They made fewer non-perseverative errors on the WCST, which indicated high flexibility and test shifting, which is a contradicting evidence for compulsivity. Furthermore, on the GO/NO-GO task, individuals who excessively use social media made more omission errors in response to the “Facebook” sign compared to traffic signs (GO condition), indicating impaired selective attention. Finally, they also showed higher subjective ratings of anxiety, depression, impulsivity, and compulsivity.

**Discussion:**

The results of this study provide evidence for impulsivity indicated by delay discounting tendency, which supports the behavioral addiction model, impaired selection attention and lack of evidence for compulsivity in excessive social media users. Further research on neurocognitive function in excessive social media users is required in order to determine whether it should be considered a behavioral addiction or an impulse control disorder.

## 1 Introduction

With the development of technology and the internet, social networks gained momentum quickly and play a central role in daily life. Social media activity has increased dramatically over the last decade. In October 2025, over 6.04 billion people worldwide used social media, representing more than 73.2% of the global population (1). This number is expected to increase to almost six billion by 2027. Internet users spend over 2 hours on social media and messaging applications every day, which is more than half an hour longer than in 2015 (1). “Facebook” leads the market, and it has increased its share from over one billion accounts to more than 3.07 billion monthly users (1).

Problematic social media use (PSMU) is defined as excessive social media use that disrupts school achievement, relationships, and well-being. It is characterized by an unhealthy level of engagement that interferes with necessary activities for optimal development (2). Social media addiction is characterized by excessive and compulsive usage that affects daily functioning and overall well-being. The term encompasses more than just spending excessive time online; it involves compulsively checking for updates, feeling anxious when offline, and experiencing negative impacts on real-life relationships and responsibilities (3).

The World Health Organization (4) pointed out the risks involved in problematic social media network use, which are similar to Internet Gaming Disorder (IGD), problematic social media use, gambling disorder, and substance use disorders. Problematic social media use has been associated with various psychiatric symptoms, including stress, anxiety, depression, obsessive-compulsive symptoms, attention-deficit/hyperactivity symptoms, and excessive alcohol use (5). It may also be associated with cyberbullying, aggression, and fear of missing out (FOMO) (6). Recent evidence showed that digital addictions are also associated with suicidal ideation. Higher level of suicidal risk is related to emotional problems, conduct problems and with poorer quality of life (7; see a review by 8).

There is little evidence for impaired cognitive and executive function in excessive social media users. There was no evidence for cognitive flexibility and inhibitory control aspects of executive function measured by performance on the Wisconsin Card Sorting Test in excessive social media users (WCST) (9). However, ratings of social media addiction were found to correlate with the categories achieved and the number of perseverative errors on the WCST (9). It should be noted that excessive use of social media is not equal to addictive use, since there are individuals who spend lots of time on social media due to professional use, and show low addiction symptoms. Finally, a study using a GO/NO-GO task with social signs found no performance impairment in problematic users compared with non-problematic users, focusing on inhibitory control mechanisms and event-related potentials (ERPs) (10). However, excessive users showed larger ERP responses to social media stimuli, indicating inefficient processing and inhibitory control mechanisms (10). Previous studies showed some evidence for impulsivity in excessive social media users (11, 12). Meshi et al. (11) found that performance in the last block of trials on the Iowa Gambling Task negatively correlated with social media scores. Although the finding is preliminary evidence for an association between excessive social media use and more deficient decision-making, there was no comparison in IGT performance between excessive social media users and non-users of social media. Similarly, Turel et al. (12) reported that an activation of the amygdala-striatal (impulsive) brain system positively correlated with one’s Facebook addiction-like symptoms, and that there was no correlation between this score and activation of the prefrontal cortex (inhibition) brain system. There was a correlation between subjective social media measures and brain activity indicating impulsivity, but no comparison with a group of non-users of social media.

Compulsivity is less well-defined and investigated than impulsivity. Few studies measure compulsive behavior using cognitive tasks in psychiatric disorders. Neurocognitive measures of compulsivity usually assess the ability to adapt behavior flexibly after negative feedback (on probabilistic reversal learning tasks) or switch attention between stimuli (on a set-shifting task like the WCST). An impaired ability to shift sets could indicate a diminished ability to disengage from repetitive acts or obsessive thoughts (13). Studies have rarely shown impaired flexibility in OCD. OCD patients showed no impairment in performance on the WCST, and no differences in WCST performance were observed in patients with OCD who were treated with fluvoxamine compared with non-treated patients (14). OCD patients showed impaired extra-dimensional shifting on the set-shifting test and impaired reversal of response set on the Go/NO-GO task (15). A study of OCD patients indicated that most neurocognitive measures did not predict treatment response, although measures of flexibility, such as an enhanced rate of perseveration errors on the WCST, predicted poor outcomes for the treatment of OCD (16). These findings indicate that tasks that measure flexibility and set-shifting, like the WCST, may be suitable to measure compulsivity.

There are different aspects of decision-making that involve increased risk-taking, impulsivity, and impaired judgment in behavioral addictions like gambling disorder (17). Problematic Internet use was associated with deficits in decision making that were assessed on the Iowa Gambling Task, Balloon Analogue Risk Task (BART), Cambridge Gambling Task, and Game of Dice Task (18). The Experiential Discounting Task is a real-time choice procedure designed to assess delay discounting, and it provides choice consequences (i.e. delays, probabilities, and monetary rewards delivered from a coin dispenser) during choice sessions; the winnings on the task are experienced directly. The standard delay discounting task used question-based measures, in which delays, probabilities, and rewards were not directly experienced (19). The BART task measures risk-taking in conditions under unknown risk (20, 21). The objective of the BART is to obtain the highest possible reward. The average adjusted number of pumps on the task represents risk-taking propensity, and a high adjusted score indicates high-risk behavior. The Cambridge Gambling Task measures risk-taking under known risks (22). Weinstein et al. (23) employed the Experiential Delay Discounting Task (EDT) and the Balloon Analog Risk Task (BART) to assess impulsivity among individuals with IGD. They found that individuals with IGD exhibited a higher tendency to delay discounting indicated by steeper slopes on the EDT, and a high propensity for risk-taking shown on the BART (23).

Few studies examined the relationships between excessive social media use, attention, and memory, usually finding a weak association. Reed et al. (24) conducted three experiments on the association between executive function and excessive social media use and found a correlation between excessive social media use and inhibitory control mechanisms, indicated by performance on the Iowa Gambling Task (IGT). In attention-switching tasks, he reported weaker correlations with attention. Furthermore, his research revealed a weaker association between attention, attention-switching tasks, and memory. Finally, his studies indicated that the relationships between excessive social media use and inhibitory control mechanisms were either exacerbated by exposure to social media or stronger when performance involved social media-related stimuli.

There is a paucity of evidence for risky decision-making in problematic social-network use. Müller et al. (25) used decision-making tasks like the Cards and Lottery Task (CLT) and the Modified Card Sorting Test (MCST), Barratt Impulsiveness Scale (BIS) in participants with excessive social media use and found attentional impulsivity but no differences in decision-making compared with those with no excessive social media use. Social media addiction symptoms in social media users negatively correlated with Gray Matter Volumes (GMVs) of the bilateral posterior insula, and this association was mediated by delay discounting, indicating impulsivity (26). Consistent with this evidence, a correlation between symptoms of problematic social media use and attentional impulsivity (but not impaired inhibitory control of cues) was reported by (27). See reviews and meta-analyses about delay discounting/risky decision-making by Cheng et al. (28) and Müller et al. (29).

Impulsivity and compulsivity are behavioral constructs controlled by brain mechanisms that are highly important for survival. When these mechanisms become dysfunctional, they contribute to a wide range of psychiatric disorders, like drug and behavioral addictions, imposing major personal, social, and economic burdens on individuals (30). The American Psychiatric Association (31) defined impulsivity as “a predisposition toward rapid, unplanned reactions to internal or external stimuli, often disregarding negative consequences”. Compulsivity, on the other hand, was characterized by “repetitive behaviors aimed at reducing or preventing anxiety or distress, rather than seeking pleasure or gratification”. Behavioral addictions lie on a spectrum between impulsivity and compulsivity, and they are often characterized as an impulse-control disorder. These behaviors are typically preceded by feelings of tension or arousal before the act is committed and they result in pleasure, gratification, or relief during the act, however, they can become addictive despite their negative consequences (32).

There is a public health concern over the effects of excessive or problematic social media use on cognitive and emotional function (6). There is a debate over whether excessive social media use should be considered a behavioral addiction. (33–35). One of the issues is whether excessive social media use is characterized by impulsivity or an impulse control disorder characterized by compulsivity. Impulsivity, executive functions, and decision-making are key features of addictive behaviors, yet research on these functions in problematic social-network use is rare, and there are inconsistent findings (25).

This study sets out to utilize neurocognitive tasks assessing impulsivity and compulsivity in excessive social media users to determine whether problematic social media use should be classified as a behavioral addiction or as included in the OCD spectrum. We utilized the EDT, which measures delay discounting, and the GO/NO-GO task, which measures inhibition using Facebook and neutral signs. Although excessive social media users show evidence for subjective compulsivity (5), few studies have used computerized cognitive tasks assessing compulsivity in them. A recent study by Müller et al. (36) used a modified WCST together with other measures of executive function in individuals with and without problematic social media, and Wegmann et al. (27) used the MCST in the context of problematic social media use. We therefore used the WCST, which measures mental flexibility and working memory, to assess compulsivity among individuals with excessive social media use compared to those without.

## 2 Hypotheses

1. Excessive social media users would show higher impulsivity compared with non-excessive social media users. These characteristics would be indicated by the steepness of the discount curve, which is the delay discounting measure of performance on the EDT, and ratings on the BIS-11 questionnaire (37).
2. Excessive social media users would show higher impulsivity compared with non-excessive social media users, as indicated by impaired inhibition of social media stimuli (Facebook signs) on the GO/NO-GO task.
3. Excessive social media users would show higher compulsivity and inflexibility compared with non-excessive social media users, which would be indicated by a high number of non-perseverative errors on the WCST and high ratings on the Y-BOCS.
4. Excessive social media users would show high ratings of anxiety on the Spielberger State-Trait Anxiety Inventory (STAI) and depression on the Beck Depression Inventory (BDI) compared with non-excessive social media users.

## 3 Methods

### 3.1 Participants

Eighty-three participants were recruited for this study. Four participants were excluded due to inadequate performance on the computerized cognitive tasks. The sample included seventy-nine participants, aged 18-37, including 16 males and 63 females. The participants were divided into two groups: one group of 34 participants classified as excessive social media users, who scored 19 or above on the Bergen Social Media Addiction Scale (BSMAS) (5) with a mean age of 23 years and 0.36 months (S.D = 2.71) and another group of 45 participants classified as non-excessive social media users, who scored lower than 19 on the BSMAS with a mean age of 25 years and 6 months (SD = 4.3). Participants were students recruited from social networks. They filled out questionnaires online. Exclusion criteria were individuals who do not use social media, are diagnosed with ADHD, or have mental or neurological illnesses. Several participants’ data is missing due to technical reasons; hence, incomplete data was retained.

### 3.2 Questionnaires

#### 3.2.1 Demographic questionnaire

The demographic questionnaire includes questions about sex, age, education, religion, marital status, urban living, frequency of use of the smartphone, frequency of use of social networks, and employment. **Table 1** shows demographic details for all participants.

**Table 1:** Demographic questionnaire ratings of the study groups (n=79).

			Group	
Variables			Excessive social media users	Non-excessive social media users
Age mean (SD)			23.03(2.7)	25.47(4.3)
Gender	Female	n	29	34
		%	85.3%	75.6%
	Male	n	5	11
		%	14.70%	24.40%
Religious	No	n	8	11
		%	23.5%	24.4%
	Yes	n	26	34
		%	76.5%	75.5%
Family status	In a relationship	n	5	7
		%	14.7%	15.6%
	Single	n	22	25
		%	64.7%	55.6%
	Married	n	7	13
		%	20.6%	28.9%
Academic education	No	n	18	21
		%	52.9%	46.7%
	Yes	n	16	24
		%	47.1%	53.3%
Frequency of use of SM last month	Less than 30 minutes	n	0	2
		%	0%	4.4%
	1 hour	n	0	6
		%	0%	13.3%
	2–3 hours	n	13	19
		%	38.2%	42.2%
	4–5 hours	n %	10 29.4%	12 26.2%
	6 hours or more	n %	11 32.4%	6 13.3%

#### 3.2.2 Bergen social media addiction scale

The Bergen Social Media Addiction Scale (BSMAS) (5) includes 6 items on a Likert scale that reflect the 6 central elements of addiction according to Griffiths (38): salience, tolerance, mood modification, relapse, withdrawal, and conflict. Items are rated on a 5-point Likert scale that ranges from 1 (very rarely) to 5 (very often), with a maximum score of 30, where higher scores indicate higher levels of social media addiction. The questionnaire had been validated with a mean Cronbach internal reliability of α=0.85 (39). In our study, the questionnaire had a Cronbach’s internal reliability of α=0.71.

#### 3.2.3 Barratt impulsiveness scale

The Barratt Impulsiveness Scale BIS-11 (37) includes 30 items on a Likert scale that range from 1 (rarely/never) to 4 (almost always/always). The BIS-11 is a 30-item self-report measure, with scores ranging from 30 to 120, where higher scores indicate higher levels of impulsivity. The questionnaire had been validated with a mean Cronbach internal reliability of α=0.79-0.83 (37). In our study, the questionnaire had a Cronbach’s internal reliability of α=0.74.

#### 3.2.4 Yale-brown obsessive compulsive scale

The Yale Brown Obsessive Compulsive Scale (40) includes ten items on a Likert scale ranging from 0 (“full control”) to 4 (“no control”). Scores range between 0 and 40, including 5 items of obsessive thoughts and 5 items of compulsive behaviors. The questionnaire had been validated with a mean Cronbach internal reliability of α=0.89 (40). In our study, the questionnaire had a Cronbach’s internal reliability of α=0.87.

#### 3.2.5 Spielberger trait and state anxiety inventory

The Spielberger Trait and State Anxiety Inventory (STAI) (41) contains forty items, twenty related to trait anxiety and twenty related to state anxiety. Scores on a Likert scale range from 1 (“not at all”) to 4 (“agree very much”). The questionnaire had been validated with a mean Cronbach’s internal reliability of α = 0.83 for Spielberger State and α = 0.88 for Spielberger Trait (41). In our study, we used the Trait anxiety inventory, which had a Cronbach’s internal reliability of α=0.91.

#### 3.2.6 The beck depression inventory

The Beck Depression Inventory (BDI) (42) is a self-report measure of attitudes and symptoms of depression (42). The inventory has twenty-one items. Scores on a Likert scale are rated from 0 to 4. The BDI has high internal consistency, with Cronbach’s internal reliability of α = 0.86 and 0.81 for psychiatric and non-psychiatric populations (43). In this study, the BDI had a Cronbach’s internal reliability of α=0.86.

### 3.3 Computerized tasks

#### 3.3.1 Experiential Delay Discounting Task (EDT)

Experiential Delay Discounting Task (EDT) by Rachlin, Raineri, and Cross (44).

The computer screen presented either 1.2 Israeli shekels that were delayed and uncertain or a lower amount than 1.2 Israeli shekels that were immediate and changeable. Participants were instructed that if they decided to cash the money, they had to press the square on which it was shown. There were 4 blocks with 15 trials in each block involving different delay times for each trial (1, 5, 10, 20 seconds).

#### 3.3.2 Analysis of the results of the experiential delay discounting task

The score of delay discounting was calculated by adding all choices and times of delay following the method described by Saville et al. (2001) (45) and Reynolds and Schiffbauer (46). K is a free parameter that indicates the steepness of the discount curve. High k-values indicate rapid discounting (e.g., 47–49). Delay discounting curves are plotted at indifference points. An indifference point was defined as the point during a block of choices when the standard- and adjusting-option amounts were of equal subjective value at a certain delay to receiving the standard option. Discounting data are well characterized by the hyperbolic model (47), notated as follows: Value = A/(1 + kD), (1) where Value represents the value of the delayed reinforcement, and A and D, are the amount of the reinforcer and length of delay to its delivery, respectively. The k is a free parameter that indicates the steepness of the discount curve. Higher k-values indicate more rapid discounting, which has been defined as more impulsive (e.g. 47–49). A number of studies have shown that patterns of discounting by delay are better characterized (i.e. are fit better) by a hyperbolic function than by exponential function (e.g. 44, 49–51), which is notated as follows: Value = Ae^−kD^ (2) where again A is the amount of reinforcer, D the delay to receiving the reinforcer, and k the free parameter indicating the steepness of the discount function. The exponential model has historically been the standard of rational choice in the field of economics (e.g. 52, 53). The findings that show better fits with a hyperbolic, non-rational model are significant in the study of impulsive behavior.

#### 3.3.3 GO/NO-GO task

Gomez et al. (54) describe the GO/NO-GO task, originally developed by Luria in the 1940s. The task requires participants to respond by pressing a button when they see a “GO” sign and not to respond when they see the “NO-GO” sign. The task measures the ability to ignore or inhibit automatic and habitual responses in favor of more deliberate actions. In this experiment, the “GO” sign was the logo of “Facebook,” and the “NO-GO” sign was arrows from a traffic sign. The experiment presented 100 trials for 1.5 seconds. The probability of sign presentation (“GO” or “NO-GO”) was equal, and they were presented in a random order. After 50 trials, the order was reversed, with the “GO” sign being the arrows from a traffic sign and the “NO-GO” sign being the logo of “Facebook.” The analysis included reaction times (RTs) and commission errors, which were falsely not pressing the button in “GO” trials, and omission errors, which were falsely pressing the button in “NO-GO” trials.

#### 3.3.4 Analysis of the results on the GO/NO-GO task

**Figure 1:**
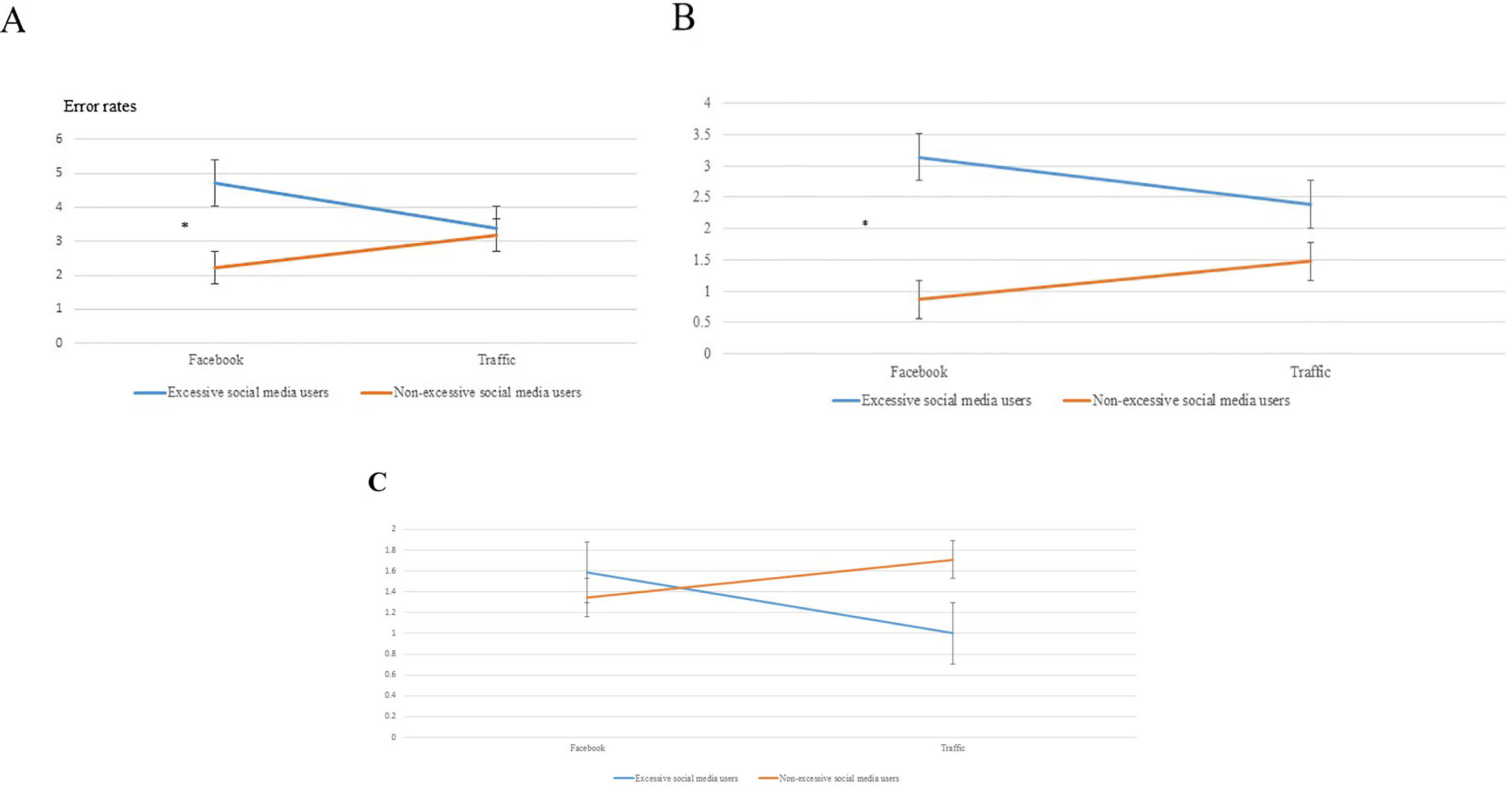
Response errors in both groups to the different signs (Facebook and traffic). **(A)** Averaged GO/NO-GO responses. **(B)** Action: GO. **(B)** shows error rates in condition GO in response to Facebook and traffic signs, **(C)** Action: NO-GO. **(C)** shows no group differences in error rates in the NO GO condition in response to Facebook and traffic signs. Non-excessive social media users show reduced RTs to Facebook signs **p<0.01. Social media users showed higher error rates in response to Facebook signs compared with non-excessive social media users *p<0.05.

The analysis included a two-way mixed repeated measures ANCOVA that examined reaction times (RTs), commission, and omission errors for two groups of participants: excessive users, and non-excessive users of social media (**Figure 1**).

#### 3.3.5 Wisconsin card sorting task

A modified short version of the Wisconsin Card Sorting-like Task (WCST) (55, 56) was used for measuring cognitive flexibility. The WCST has 64 response cards and 4 stimulus cards. The stimulus cards were presented in a standard left-to-right order, while response cards were presented one by one in a specific criterion (color, shape, or number). In the sorting task, the response card has to correspond to a feature of the target card. After a sequence of 10 correct responses, the sorting criterion changes, and a new sorting criterion must be detected. The task included 64 trials, and for each participant, the following indices were registered: (a) number of sets completed, (b) number of maintaining set failures, (c) number of perseveration errors (set-shifting failures), and (d) number of non-perseveration errors.

#### 3.3.6 Analysis of the WCST

The analysis included one-way ANCOVAs for the 4 indices: (a) number of completing sets, (b) number of maintaining set failures, (c) number of perseveration errors (set-shifting failures), and (d) number of non-perseveration errors.

## 4 Procedure

The participants were University participants, who were recruited by social networks. The university approved the research protocol and ethical approval, marking the start of data collection. The questionnaires were filled out online using Google Forms on the computer, and afterwards, in the same session, the participants were assessed individually on the computerized tasks in the laboratory. The participants received participation vouchers as part of their degree requirements.

### 4.1 Ethical approval

The study was approved by the Institutional Review Board (IRB, Helsinki committee) of the University, issue number AU-SOC-AW-20230530 from 30/5/2023. An informed consent form was signed by all participants.

### 4.2 Statistical and data analysis

Results were analyzed using version 21 of the Statistical Package for Social Science (SPSS) (IBM Corp. Armonk, NY, USA). Pearson’s chi-squared test was used to compare demographic factors such as sex, age, education, religion, marital status, urban living, frequency of use of the smartphone, frequency of use of social networks, and employment. Group comparisons were done using one-way ANCOVA for the BIS-11, YBOCS, STAI, and BDI questionnaires and the EDT, WCST, and GO/NO-GO tasks. Pearson’s correlation tests were used for associating questionnaire ratings and performance on the cognitive tasks.

### 4.3 Power calculations

Power calculation of the ANOVA model was conducted using G*Power 3.1.9.7 (57, 58) based on ANCOVA and two-way mixed-design repeated-measures ANCOVA in accordance with the relevant literature (11). For the ANCOVA, an expected effect size of 0.3 with a standard statistical power of 0.8 was selected, yielding a minimal sample size of 67 participants. For the two-way mixed design repeated measures ANCOVA, an expected effect size of 0.25 and a standard statistical power of 0.8 requires 2 groups of participants with a minimal sample size of 34 each. For within-group comparisons, an expected effect size of 0.25 with a standard statistical power of 0.8 was selected, including 2 groups of participants with a minimal sample size of 34 each.

## 5 Results

### 5.1 Descriptive statistics

Preliminary analysis was performed on the factors of age, education, marital status, and level of religiosity. A comparison between groups using one-way ANOVA showed that excessive social media users were younger than the non-excessive social media users, F (1, 78) = 106.5, p=0.01. The mean age of excessive social media users (M = 23.03, *SD* = 2.71) was lower than those who do not use the media excessively (M = 25.47, *SD* = 4.3). There was no significant difference in education, F (1, 78) = 0.95 p= N.S and marital status, F (1,78) = 0.34 p= N.S. Subsequent analyses used age as a covariate for the questionnaires and computerized tasks.

### 5.2 Questionnaire ratings

**Table 1** shows demographic data for all participants.

**Table 2** shows comparisons between groups based on questionnaire ratings.

**Table 2:** A comparison between excessive and non-excessive social media users in questionnaire ratings and cognitive tasks’ performance (*N = 79).*.

Questionnaire	Excessive social media use (SD)Mean	Non-excessive social media use Mean (SD)	F	*p*	Effect Size ηp^2^
BSMAS	20.91(2.06)	13.73(3.23)	F(1,76) =127	0.001	ηp^2^=.624
BIS-11	62.47(8.72)	56.2(6.97)	F(1,76) =8.7	0.004	ηp^2^=.103
YBOCS total	20.82 (5.18)	16.93(4.4)	F(1,76) =9.11	0.003	ηp^2^=.107
BDI	29.47(7.22)	24.47(4.25)	F(1,76) =9.62	0.003	ηp^2^=.11
STAI	42.41(9.68)	33.75(8.36)	F(1,76) =11.5	0.001	ηp^2^=.131
EDT K	-0.09(0.09)	-0.04(0.1)	F(1,75) =4.55	0.04	ηp^2^= .06
WCST-number of completed sets	4.03(0.96)	4.02(1.05)	F(1,74) =-0.046	0.83	ηp^2^=.001
WCST- number of maintenance of set failures	0.5(0.76)	0.42(0.69)	F(1,74) =-0.23	0.636	ηp^2^=.003
WCST-number of perseverative errors	6.51(4.22)	5.75(2.23)	F(1,73) =0.71	0.40	ηp^2^=.01
WCST-number of non-perseverative errors	4.83(2.16)	6.38(4.02)	F(1,73) =4.5	0.037	ηp^2^=.058

BSMAS, Bergen Social Media Addiction Scale; BIS-11, Barratt Impulsiveness Scale; YBOCS, Yale Brown Obsessive Compulsive Scale; BDI, Beck Depression Inventory; STAI, Spielberger Trait Anxiety Inventory; EDT, Experiential Delay Discounting Task; K, discount parameter; WCST, Wisconsin Card Sorting Task.

Several participants’ data is missing due to technical reasons, hence incomplete data was retained.

Only few participants did not complete the tasks (n=4) and three did not complete the questionnaires and these were excluded from analysis.

**Figure 2:**
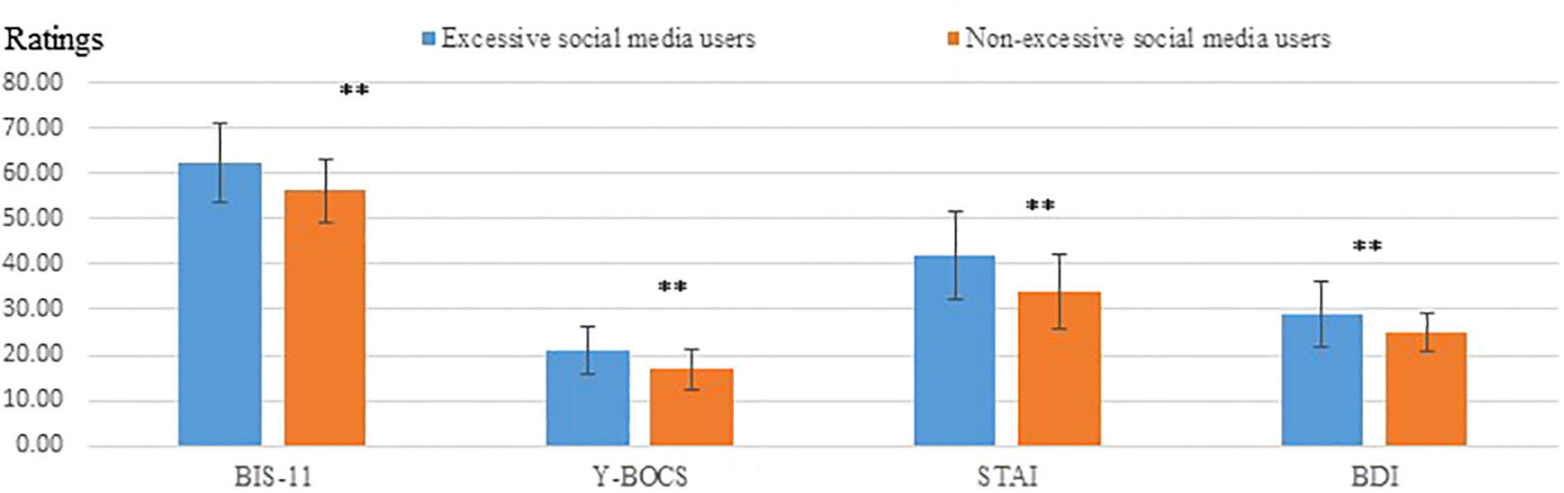
Questionnaire ratings of all participants (N=79). BIS-11, Barratt Impulsiveness Scale; YBOCS, Yale Brown Obsessive Compulsive Scale; STAI, Spielberger Trait Anxiety Inventory; BDI, Beck Depression Inventory. Excessive social media users scored higher on BIS-11, Y-BOCS, STAL, and BDI questionnaires compared with non-excessive social media users, **p<0.01.

Participants with excessive social media use scored higher than those with non-excessive social media use on the BIS-11 impulsivity questionnaire using ANCOVA with age as a covariate, F (1, 76) = 8.7, p=.004; ηp^2^ = .103. Participants with excessive social media use scored higher than those with non-excessive social media use on the YBOCS questionnaire using ANCOVA with age as a covariate, F (1, 76) = 9.11, p=.003; ηp^2^ = .107. See **Figure 2** for questionnaire ratings in all participants.

#### 5.2.1 Anxiety and depression questionnaires

An ANCOVA compared participants with excessive social media use to participants with non-excessive social media use, with age as a covariate, and found a group difference in Spielberger trait anxiety (F (1, 76) = 11.5, p=0.001, ηp^2^ = .131). Participants with excessive social media use had higher trait anxiety scores (M = 42.41, SD = 9.68) compared with participants with non-excessive social media use (M = 33.75, SD = 8.36), see **Figure 2**.

An ANCOVA compared participants with excessive social media use to participants with non-excessive social media use, with age as a covariate, and found a group difference in BDI scores (F (1, 76) = 9.62, p=0.003, ηp^2^ = .112). Participants with excessive social media use scored higher on the BDI (M = 29.47, SD = 7.22) compared with non-excessive social media users (M = 24.47, SD = 4.25), see **Figure 2**.

### 5.3 Cognitive tasks

The first hypothesis—excessive social media users would show higher impulsivity compared with non-excessive social media users, as indicated by delay discounting measures of performance on the EDT.

#### 5.3.1 EDT

**Figure 3:**
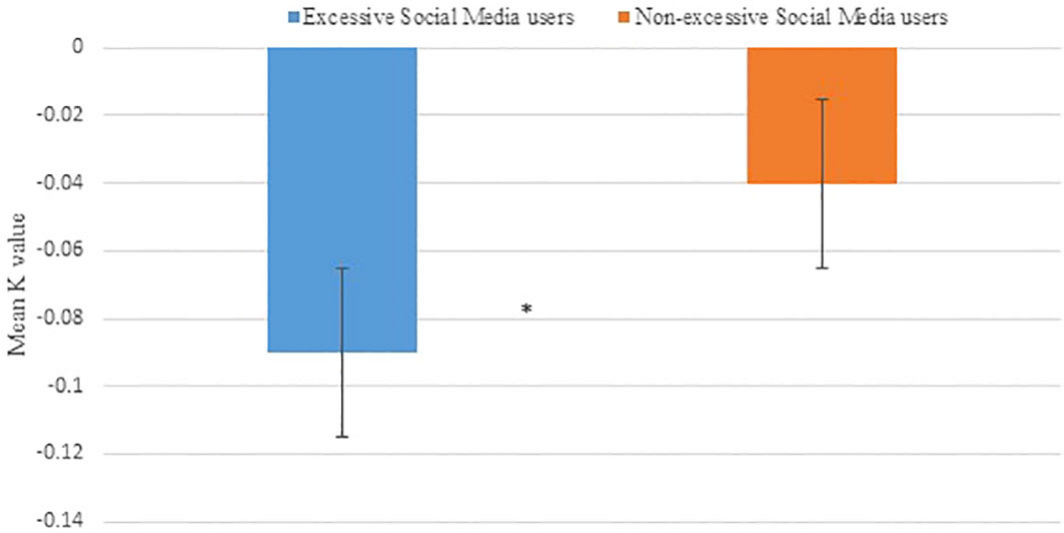
Mean K value and S.D on the experiential delay discounting task (EDT) in excessive and non-excessive social media users (N=79). The total mean K score of excessive social media users was lower than non-excessive social media users (*p<0.05), suggesting that they had higher rates of delay discounting.

There was a significant difference in the K value (slope of the delay discounting curve)between participants who excessively used social media and those who did not, with non-excessive use of social media as determined by an ANCOVA with age as a covariate, F (1, 75) = 4.55, p = 0.04, ηp^2^ = .06), indicating delay discounting. The total mean K score of the excessive social media use was -0.1 (S.D. = 0.1), and the total mean score of the non-excessive social media use was -0.04 (S.D. = 0.1), suggesting that the excessive social media users had steeper slopes and were more impulsive. **Figure 3** indicates differences in average group performance on the delay discounting task between excessive and non-excessive social media users.

#### 5.3.2 GO/NO-GO

**Figure 4:**
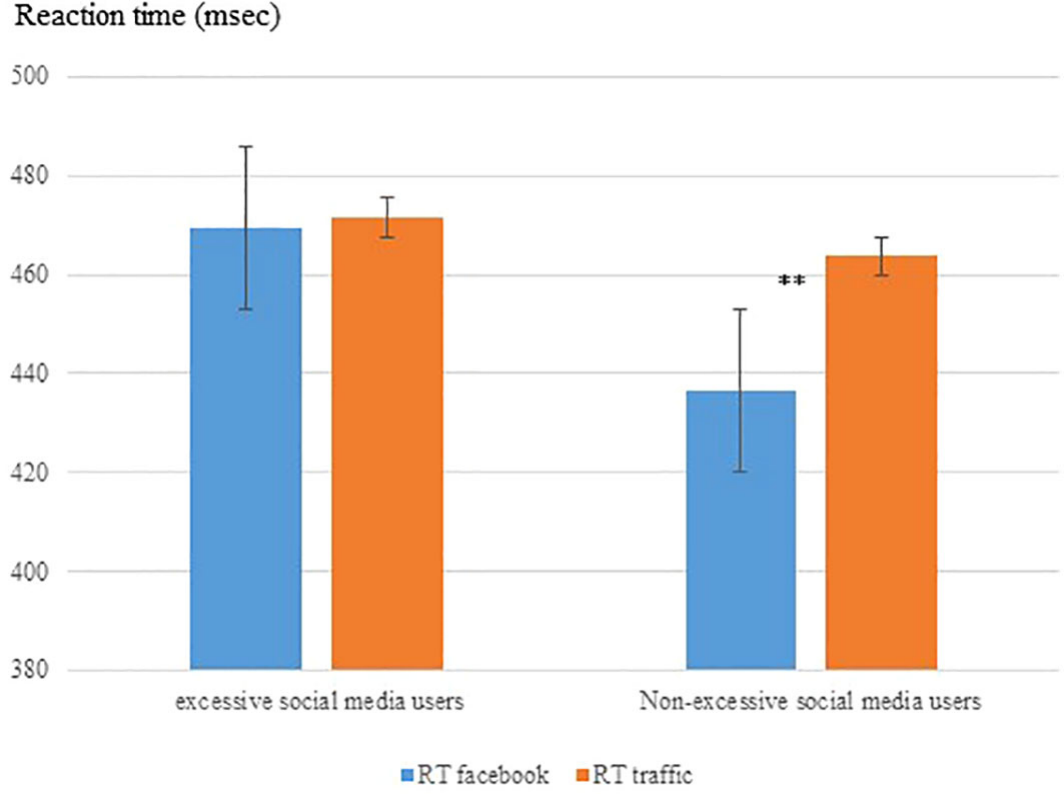
Reaction time on the GO/NO-GO task as a function of sign type and group (excessive and non-excessive users of social media), n=76. Non-excessive social media users show reduced RTs to Facebook signs **p<0.01.

A two-way mixed design repeated measures ANCOVA was conducted to explore the effect of group (excessive vs. non-excessive social media users) and sign type (Facebook vs. traffic sign) on RTs with age as a covariate. Results revealed an interaction between group and sign type (F (1, 75) = 4.02, p = .04, ηp^2^ = .05) but there was significant group effect (F (1, 75) = 1.32, p=.25, ηp^2^ = .02) or sign type effect (F (1, 75) = 1.43, p=.23, ηp^2^ = .02). *Post-hoc* analysis revealed that participants with non-excessive social media use were faster to respond to “Facebook” signs compared to traffic signs (F (1, 75) = 9.95, p = .002, ηp^2^ = .12). For participants with excessive use of social media, there was no difference in RTs between signs (F (1, 75) =.00, p= .99, ηp^2^ = .00). **Figure 4** shows response times in both groups to the different signs. The faster response to “Facebook” signs in non-excessive social media users may indicate a response bias.

#### 5.3.3 Analysis of errors

A mixed-measures ANOVA of the error rates was conducted to explore the effect of group (excessive vs. non-excessive social media users) and sign type (Facebook vs. traffic sign) on errors with age as a covariate. Results reveal no effect of group (F (1, 75) = 1.86, p=.18, ηp^2^ = .02) or sign type (F (1, 75) = 1.60, p=.69, ηp^2^ = 4.81×10-4). There was an interaction between group and sign type after a Bonferroni correction (F (1, 75) = 5.32, p = .024, ηp^2^ = .016). *Post-hoc* analysis revealed that participants with excessive social media use made more omission errors in response to “Facebook” signs compared to non-excessive social media users (t (96) = -2.26, p = .026, ηp^2^ = .12), but not in response to traffic signs (t (96) = -0.197, p = 0.86). The higher number of omission errors to “Facebook” signs in the GO condition in excessive social media users may indicate impaired attention and not inhibition, since participants are required to respond to the sign (GO) and not inhibit their responses (NO GO).

#### 5.3.4 The Wisconsin card sorting task

An ANCOVA comparing participants with excessive social media use to participants with non-excessive social media use, with age as a covariate, showed:

1. There was no group difference in the number of completed sets (F (1, 74) = .046, p=.831, ηp^2^ = .001).
2. There was no group difference in maintaining set failures (F (1, 74) = .226, p=.636, ηp^2^ = .003).
3. There was a group difference in non-perseverative errors (F (1, 73) = 4.5, p=.037, ηp^2^ = .058). Participants with excessive use of social media made fewer non-perseverative errors (M = 4.84, S.D = 2.16) compared with participants who are non-excessive social media users (M = 6.38, S.D = 4.02).
4. There was no group difference in perseverative errors (F (1, 73) = .711, P = .402, ηp^2^ = .01).

**Figure 5:**
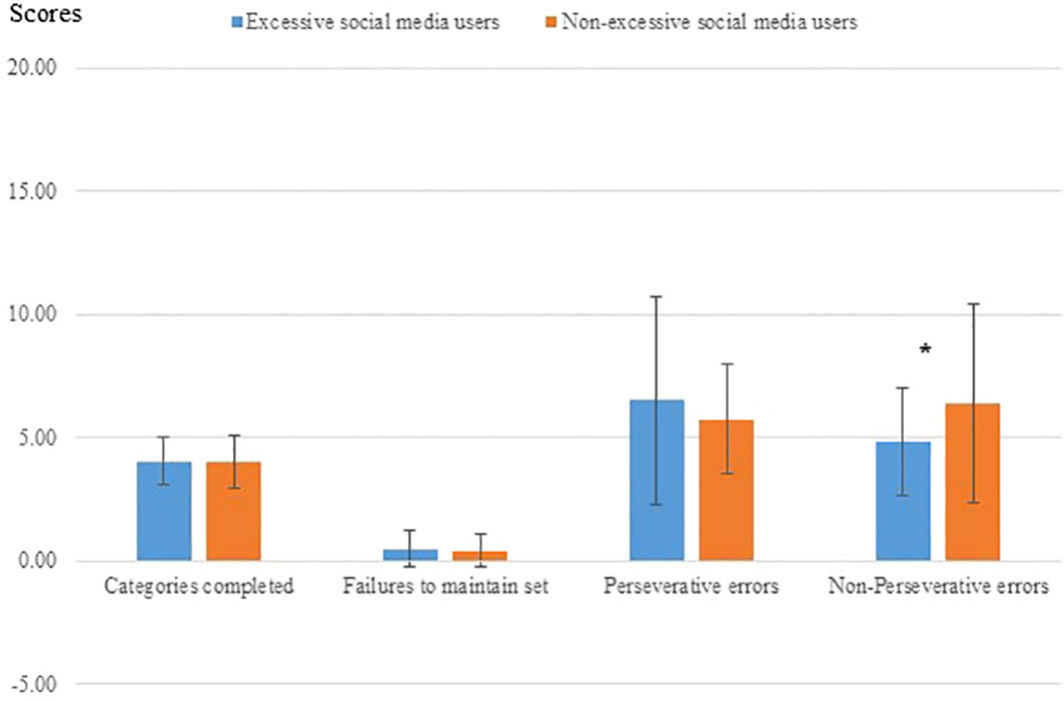
Performance on categories of the WCST in excessive and non-excessive social media users (n=79). Excessive social media users made fewer non-perseverative error compared with non-excessive social media users *p<0.05.

**Figure 5** shows group differences in the number of completed sets, maintaining set failures, perseverative errors, and non-perseverative errors.

Correlations between questionnaire ratings and performance on cognitive tasks.

There were no correlations between the BIS-11, YBOCS questionnaires, and the EDT, GO/NO-GO, and WCST tasks. **Table 3** shows correlations between questionnaires and cognitive tasks.

**Table 3:** Pearson correlations between BIS-11 and YBOCS questionnaire means and EDT, WCST, GO\NO-GO tasks (*N=79*).

Variables	1	2	3	4(1)	4(2)	4(3)	4(4)	5(1)	5(2)	5(3)	5(4)
BIS-11	–										
YBOCS	.576**	–									
K value	.018	-.136	–								
WCST											
Factor 1	-.071	-.002	-.087	–							
Factor 2	.162	.089	.186	-.651**	–						
Factor 3	.057	.068	-.069	-.574**	.161	–					
Factor 4	-.07	-.094	.062	-.656**	.256*	.235*	–				
GO\NO-GO											
Factor 1	.133	.06	-.056	-.171	.314**	-.104	.126	–			
Factor 2	.095	.06	-.147	-.152	.211	-.085	.217	.808**	–		
Factor 3	.183	.12	-.074	-.19	.288*	.02	.165	.760**	.669**	–	
Factor 4	.057	.128	-.122	-.171	.174	-.041	.23*	.499**	.795**	.635**	–

*p<0.05., **p<0.01.

WCST, Wisconsin Card Sorting Task; Factor 1, Number of completing sets; Factor 2, Maintaining set failures; Factor 3, Perseverative errors; Factor 4, Non-Perseverative errors.

GO\NO-GO task: Factor 1, RT Facebook; Factor 2, RT sign; Factor 3, SD Facebook; Factor 4, RT sign.

#### 5.3.5 Sex differences

There were no sex differences in BSMAS scores, F (1,78) = 2.52, p=0.117.

## 6 Discussion

This study investigated neurocognitive function in excessive and non-excessive social media users using a task that measured impulsivity, the EDT, and a task that measured test-shifting and executive function, the WCST. The findings show evidence for impulsivity in excessive social media users, indicated by a high discounting tendency on the EDT task and high scores on the BIS-11 questionnaire that measures impulsivity. These findings demonstrate direct evidence for impulsivity in excessive social media users. Previous evidence for impulsivity in excessive social media users is based on a negative correlation between social media scores and performance on the Iowa Gambling Task (11) and a correlation between Facebook addiction-like symptoms and activation of the amygdala-striatal (impulsive) brain system (12). These studies showed no differences between excessive social media users and control participants in performance on the respective tasks. Both pathological gamblers and individuals with substance use disorders quickly discount rewards (59). Our study provides evidence for high delay discounting among excessive social media users, which is an indication of impulsivity similar to gambling disorder and IGD. Our results are also compatible with those reported by Delaney et al. (60), who found that those addicted to “Facebook” discounted delayed rewards more quickly than control participants, suggesting that they may be more impulsive than non-addicted individuals. These findings support the behavioral addiction model, which includes impulsivity as a core component.

Impulsivity is the inclination of an individual to act upon urges or the inability to plan or consider various options before making a decision (61). Impulsivity was also defined as a predisposition to look for excitement or adventure, impatience, and a lack of the ability to foresee the consequences of one’s actions, and to behave without control or inhibition (62, 63). Research demonstrated that impulsive traits such as positive and negative urgency and lack of perseverance are associated with problematic social media use (64). On the other hand, “perseverative errors” on the Modified Card Sorting Test may be indicative of compulsivity (25). Young smartphone users who spend more time on social media reported addictive measures that correlated positively with impulsivity and negatively with self-efficacy (65). Motivations for using social media vary, including handling boredom, increasing social rewards (“likes”), social comparisons, social connections, and high expectations of having a large network (6).

The study showed lower rates of non- perseverative errors on the WCST, which provides a negative indication for compulsivity in excessive social media users. Non-perseverative WCST errors occur when the participant, during set shifting, does not discriminate between errors related to the efficient test of hypotheses (‘efficient errors’) or makes random failures to maintain set (‘random errors’) (66). Since participants with excessive social media use showed lower rates of non-preservative errors, this is evidence for highly efficient hypothesis shifting and set maintenance, contrary to our prediction of neurocognitive impairment in this group. This evidence stands in contrast to their high scores on the YBOCS (Yale-Brown Obsessive Compulsive Scale), which measures compulsivity. Furthermore, the subjective measures of compulsivity did not correlate with the categories indicating perseverance and flexibility on the WCST task.

As mentioned earlier, there are hardly any cognitive tasks that reliably measured inflexibility and compulsivity in various clinical conditions. Even in a clinical population of OCD patients, the results are mixed. OCD patients showed no impairment in performance on the WCST, and no differences in WCST performance were observed in patients with OCD who were treated with fluvoxamine compared with non-treated patients (14). Our study made an attempt of assessing flexibility using the WCST in a normal non-clinical population of excessive social media users. The inability to prove evidence for inflexibility on the WCST may be due to the tasks’ insensitivity to detect inflexibility in individuals with behavioral addictions. This conclusion is supported by a recent review by Morris and Voon (67), who provided evidence that tasks measuring flexibility like the WCST showed mixed results in behavioral addictions. Although there is limited, evidence for higher perseveration errors on the WCST in binge eating disorder patients, the WCST has yielded conflicting results among patients with pathological gambling. Increases in non-perseverative errors have been reported in Pathological Gamblers performing on the WCST, suggesting that the observed impairments may not be specific to set-shifting but more of a broader cognitive dysfunction (68). Our findings show that individuals with excessive social media use are actually more efficient at set shifting and set maintenance despite their compulsivity. Another possible reason for the lack of impairment in compulsivity is that the task was not associated with behavior-specific stimuli (69). A single study on compulsive sexual behavior paired a behavior-specific exposure with the WCST, and found no difference at baseline, but worse WCST performance in those with compulsive sexual behavior after an erotic video exposure (70). It is possible that in order to show a lack of flexibility there is a need for a condition-related exposure, such as the one we used with Facebook signs on the Go/No-Go task.

There are often discrepancies between subjective reports of impulsivity/compulsivity and computerized set-shifting tasks. A recent study using questionnaires assessing subjective ratings of impulsivity or compulsivity and tasks’ performance in compulsive buyers found that there was little correlation between subjective questionnaires and cognitive tasks, suggesting that these examine different cognitive mechanisms (71). Self-reports often measure subjectively perceived, daily-life behaviors, while tasks measure specific, context-dependent cognitive abilities. Individuals may report high impulsive symptoms while performing normally on cognitive tasks in the laboratory, since questionnaires cover wider behavioral contexts than specific cognitive abilities (71).

There are important reasons for these discrepancies which include differences in context. Computerized tasks measure narrow aspects of cognitive control in a low-stakes environment, whereas subjective reports reflect behavior in complex, high-emotion, real-world situations. Questionnaires can be influenced by self-awareness or social desirability, whereas computerized tasks provide objective measurements of cognitive processes. Finally, while subjective impulsivity/compulsivity relates to how individuals process errors, this does not always translate into lower performance on cognitive tests. Impulsivity and compulsivity are conceptualized as distinct, but potentially overlapping dimensional traits. A person might report high compulsive tendencies (e.g., anxiety-driven repetition) but shows the cognitive flexibility to perform well on a set-shifting test, or vice versa. (72). The discrepancy often highlights that subjective experience and specialized cognitive performance tests measure different, although related, aspects of impulsivity and compulsivity (71–73).

The GO/NO-GO task performance showed no evidence for response inhibition impairment in excessive social media users. Such impairment would be indicated by more commission errors in response to the “Facebook” sign during the NO-GO trials. Excessive social media users made more omission errors in the GO condition in response to Facebook signs. These findings may indicate an impairment in selective attention or working memory rather than impaired response inhibition. These findings are in accordance with the results of Reed et al. (24) who reported a weak association between excessive social media use and attention. Reed et al. (24) reported weaker correlations between excessive social media use and attention in attention-switching tasks. His studies also showed that the relationships between excessive social media use and inhibitory control mechanisms were either exacerbated by exposure to social media or stronger when performance involved social media-related stimuli, which is contrary to our results that did not show commission errors in response to “Facebook” signs.

It is important to distinguish between response inhibition and general attention deficits. Executive function involves three components: inhibitory control (selective attention, cognitive inhibition, and self-control), working memory, and cognitive flexibility. Inhibitory control includes the ability to control attention in order to avoid internal urges, and to act appropriately; without inhibitory control, we would act upon impulses and old habits (74). Inhibitory control of attention allows us to selectively attend, focus on what we select and suppress attention to other stimuli (75). Inhibition therefore plays an important role in selective attention, i.e., the deployment of attentional focus on task relevant features of the environment. The ability to sustain attention is maintained by higher-order executive functions such as inhibition and working memory.

Lewin et al. (76) asserted that a positive correlation exists problematic social media use and impulsivity, encompassing attentional impulsivity and impulsive decision-making. However, most impulsive action studies that replaced traditional neutral stimuli with social-media-specific stimuli (e.g., GO/NO-GO task) failed to show such a relationship. In contrast, all studies of impulsive choice that used the Delay Discounting Task, which measures how much people evaluate rewards over time, or the Iowa Gambling Task which assesses decision-making under uncertainty, revealed a relationship with problematic social media use. Problematic social media use correlated with impaired decision-making on the Iowa gambling task, similarly to substance abusers (11). However, only the last block of trials showed a negative correlation between Facebook addiction scores and performance on the IGT. This data is weak evidence of impulsivity since it is based on correlation rather than group differences. Problematic social media users also displayed high self-disclosure, posting on social media sites while disregarding long-term risks (77). Studies that measured risk-taking behavior using the Balloon Analogue Risk Task, a psychological test where participants inflate a virtual balloon to earn money while risking its explosion, showed contradictory results. There was no association with problematic social media use (78), except for a study in which problematic social media correlated with a greater affinity for high-risk situations under both risk and ambiguity, in the absence of learning (79).

A major issue in this study is the gender imbalance, since the majority of participants (80%) were women. Women showed better performance on tasks that measure impulsivity, like the EDT. A meta-analysis of 33 studies of delay discounting, revealed that women have a better ability to delay gratification, although this advantage had a small effect size presumably due problems in the precision of the instruments and small sample sizes (80). Furthermore, this sex difference emerges early in development and it is moderated by hormonal changes during the menstrual cycle (81). In our study, although the majority of our sample consisted of women, still excessive social media users showed an impairment in delay discounting. It is unlikely that it results from a sample bias- if there was a majority of males, the effect of delay discounting would be the same. Furthermore, males fail to inhibit a pre-potent response on the Go/No-Go task whereas women make more errors on the Stop Signal Task than men (82). In our study, the performance on the GO/NO-GO task showed no evidence for response inhibition impairment in excessive social media users. It is plausible that if there was a majority of male participants in our study, an impairment on the GO/NO-GO task would be detected. However, these sex differences are more evident in childhood, and they are inconsistent in adults (82).

Another limitation is the use of a lenient cut-off score of 19 on the BSMAS in order to identify people who may be addicted or at high risk of problematic social media use. The score of 19 typically indicates “at-risk” behavior, and it was derived from a study (39) using latent profile analysis to identify at-risk youth. It is regarded as a lenient threshold compared to higher cut-offs like 24, employed by Luo et al. (83). A cut-off score of 19 may be overestimating prevalence compared to clinical interviews which suggest a higher threshold.

The higher scores on questionnaires that measure depression (BDI) and trait anxiety (STAI) in excessive social media users are compatible with the literature. Young adults who use social media excessively feel more socially isolated than those with low social media use (84). Anxiety correlated with the intensity of “Facebook” use (85). Among college students, heightened social anxiety and low self-esteem result in overusing the internet and social network sites due to fear of negative evaluation from others (86). In a meta-analysis, depressive symptoms were correlated with time spent using social media and intensity of social media use (87). A cost-benefit analysis of using social media yields the costs of increased exposure to harm, social isolation, depression, and cyberbullying. The benefits are increased self-esteem, social support, social capital, testing safe identity, and a higher chance for self-disclosure (88).

Initially, behavioral addictions have been characterized as an impulse-control disorder. These behaviors are characterized by experiencing tension or arousal before engaging in the act, followed by feelings of pleasure, gratification, or relief during the act and they can become addictive despite their negative consequences (32). There is a current debate about whether to classify conditions like compulsive sexual behavior (CSB) and compulsive buying/shopping behavior (BSD) as a behavioral addiction rather than as an impulse control disorder. The lack of rigorous scientific evidence raised our doubts as well as others’ about whether compulsive sexual behavior should be considered a behavioral addiction (89). The ICD-11 currently considers problematic pornography use as a type of compulsive sexual behavior disorder, categorized as an impulse control disorder, not a behavioral addiction. Compulsive buying/shopping disorder (BSD) is currently considered in the ICD-11 as another specified impulse control disorder, not a behavioral addiction. Examination of the research in this area does not indicate strong evidence for an impairment of executive function in BSD, leading Thomas et al. (90) to conclude that future research is required to systematically examine affective and cognitive interactions in compulsive BSD.

The evidence examined so far regarding excessive social media use presents inconclusive outcomes. The early studies showed correlations of symptoms of excessive social media use and performance or brain activity (11, 12, 26). Recent research demonstrated that the severity of symptoms associated with social media use correlated with attentional impulsivity, but not with executive function or inhibitory control of social media-related cues (27). Furthermore, evidence indicated attentional impulsivity in individuals with excessive social media use (25). The studies that examined the relationships between excessive social media use, attention, and memory usually found a weak association (24). Our results indicate evidence for steeper slopes of delay discounting in excessive social media users, supporting the argument for impulsivity. Taken altogether, the research reviewed so far implies that in the area of problematic social media use, there is mixed and little convincing evidence for impairment in executive function and impulsivity. This evidence lacks consistent neuro-cognitive findings of impulsivity, which is an important component of behavioral addictions (see 91).

The current study has important implications for the understanding of the use of digital technology, and in particular of social networks, social interaction, and influencing public opinion. In the current digital age, in which social networks penetrate all aspects of life, from personal aspects to political and economic aspects, research in this field provides significant insights. Understanding the issue of compulsion and impulsivity among users who overuse social networks is essential to increasing awareness of the consequences of excessive use of social networks. The use of social networks is a tool where users can receive immediate rewards conveniently and available anywhere and at any time (92), thereby stimulating the person’s preoccupation with online-communication applications, increasing the urge to use it, and to stay online on these platforms for an extended period of time, with diminished control over use (93). The impulsive person has difficulty resisting these rewards and temptations, and thus may use social networks excessively. Studies show that impulsivity may be a risk factor for the development and maintenance of excessive social media use (64). Young smartphone users, who spent more time on social networking sites reported higher addictive tendencies, which positively correlated with impulsivity (65). Furthermore, the results of our study may provide some evidence for the ongoing debate whether excessive use of social networks may also be regarded as a behavioral addiction. The gender dominance of women in the sample highlights the need for comparing the effects of social media use between men and women, to include more demographically diverse samples, and to examine additional variables, such as age, education, and socioeconomic status. Future studies could also compare the different platforms, analyze user characteristics, emotional content, and social interactions.

## 5 Limitations

This is a cross-sectional study, so no inferences about causality can be made. Secondly, the majority of participants (80%) were women, and there is evidence that they perform better on cognitive tasks measuring impulsivity, such as delay discounting, but the effect is moderated by hormonal changes during the menstrual cycle (81). Third, the BSMAS may create a potential classification bias since it includes the 6 components of addiction. There are other questionnaires assessing dimensions of social networking use, but they do not assess components of excessive or problematic social media use, which are important in order to determine impulsivity and compulsivity. Fourth, there was no correlation between subjective reports of impulsivity or compulsivity and cognitive tasks’ performance, suggesting that these examine different cognitive mechanisms. Perhaps tasks that utilize social media stimuli are better suited to detect impulsivity and compulsivity. Finally, assessing compulsivity using cognitive tasks is problematic, and very few studies have shown cognitive impairments in clinical or subclinical populations like excessive users of the internet.

## 6 Conclusions

The results of this study support previous evidence of impulsivity, indicated by high delay discounting tendency on the EDT, and impaired selective attention on the GO/NO-GO task, and suggest that compulsivity is not evident in excessive social media users, as indicated by fewer non-preseverative errors on the WCST. High delay discounting tendency supports the evidence for impulsivity and the behavioral model of addiction. The results of this study also showed discrepancies between subjective reports of impulsivity/compulsivity and performance on computerized tasks. Individuals may report high impulsive and compulsive symptoms while performing normally on cognitive tasks in laboratory, since questionnaires cover wider behavioral contexts than specific cognitive abilities. Further research is required on neurocognitive function in excessive social media users to facilitate the debate on whether it should be considered as a behavioral addiction or merely an impulse control disorder.

## Data Availability

The raw data supporting the conclusions of this article will be made available by the authors, without undue reservation.
